# Effectiveness and safety of traditional Chinese medicines for non-alcoholic fatty liver disease: Protocol for systematic review and meta-analysis

**DOI:** 10.1097/MD.0000000000020699

**Published:** 2020-06-19

**Authors:** Manman Qin, Fuqiang Yuan, Jiankun Ren, Zhenhai Chi

**Affiliations:** a Jiangxi University of Traditional Chinese Medicine, Wanli District, Nanchang, Jiangxi; b Henan Vocational College of Nursing, Anyang, Henan; c Department of Acupuncture-Moxibustion, the Affiliated Hospital of Jiangxi University of Traditional Chinese Medicine, Nanchang, Jiangxi China.

**Keywords:** meta-analysis, non-alcoholic fatty liver disease, systematic review, traditional Chinese medicines

## Abstract

**Background:**

Previous reviews indicate that the effect of Traditional Chinese medicines (TCM) on non-alcoholic fatty liver disease (NAFLD) remains uncertainty. The study results published in the past 8 years may change this situation, but there is no updated systematic review. Therefore, we designed this study to systematically evaluate the effectiveness and safety of TCM in the treatment of NAFLD.

**Methods and analysis:**

We will search nine online databases from inception to October 01 2019, and the language will not be restricted on included trials. Randomized controlled trials that included patients with NAFLD receiving TCM therapy versus a control group will be included. Two researcher will perform independently the selection of studies, risk of bias assessment and data extraction. We will use the RevMan V.5.2 software with fixed effects model or random effects model according to the heterogeneity test to conduct the data synthesis. We will present the dichotomous data and the continuous data with risk ratios with 95% CIs and weighted mean differences or standardized mean differences with 95% CIs. The Grading of Recommendations Assessment, Development and Evaluation (GRADE) system will be used to evaluate the evidence quality with low risk, unclear risk, and high risk.

**Results:**

This study will demonstrate an evidence-based review of TCM for NAFLD.

**Conclusion:**

The study will provide clear evidence to assess the effectiveness and side effects of TCM for NAFLD.

## Abstract 2

Supplemental Digital Content is available in the text

**Key Points**

- We use the GRADE system to evaluate the evidence quality, which would help clinicians and patients with NFLD decide whether to choose TCM therapy.
- Due to the use of multiple clinical results in published trials, it may not be possible to perform a summary analysis of all included studies; however, group analysis will be performed based on different results.

## 1 Introduction

Non-alcoholic fatty liver disease (NAFLD) is one of the most common chronic liver diseases in the world. Its progressive stage is liver inflammation and fibrosis, which is called non-alcoholic steatohepatitis (NASH). NASH can cause liver cirrhosis, liver failure and liver cancer.^[1,2]^ Due to the large-scale spread of obesity, especially in Western countries, the overall prevalence of NAFLD worldwide continues to increase and is currently estimated to be 24%.^[3]^ It is worth noting that 8% to 19% of NAFLD patients were found to be wasting or non-obese in Asia.^[4,5]^ NAFLD has become the second leading cause of liver transplantation in the United States. In general, non-progressive NAFLD is asymptomatic and the drug can be cured, while progressive NASH is difficult to treat. Most drugs on the market, such as vitamin E, only improve hepatic steatosis and inflammation during the treatment of NAFLD,^[6]^ but have little effect on progressive fibrosis.^[7]^ Various clinical trials used to test NASH candidate modern drugs have failed to reach the primary end point or have limited therapeutic effects, such as obeticholic acid.^[8]^ Drugs such as nuclear receptor agonists (omedicholic acid, GFT505, elafibranor), insulin sensitizers (gliglitazone, pioglitazone, metformin), and glucagon-like peptide-1 receptor agonists are still during the development process.^[9–11]^ It takes up to 3 years to register promising anti-NASH drugs. To date, the US Food and Drug Administration (FDA) has not approved any drugs to treat NASH. Currently, weight loss can only be effective through weight loss surgery treatment or through healthy lifestyle / dietary methods and / or non-pharmaceutical management of physical activity.^[12,13]^ Therefore, the development of drugs for the treatment of NAFLD, especially for the treatment of incurable disease NASH, is an unmet medical need.

Traditional Chinese medicine (TCM) is the main source of natural medicine and herbal products, and an indispensable resource for the development of liver protection drugs. Although large-scale randomized controlled trials (RCTs) have no convincing evidence to support the treatment effect of TCM, a recent survey shows that 20% to 30% of patients in Indonesia use traditional drugs to treat various diseases,^[14]^ while some Asian countries the use of TCM has increased in recent years. Another survey showed that the use of herbal medicine to treat chronic liver disease as a complementary and alternative medicine was similar, at respectively.^[15]^ In another systematic meta-analysis with 5904 patients from 62 RCTs, TCM reduced alanine aminotransferase (ALT), aspartate aminotransferase and radiation steatosis, thereby benefiting the treatment of NAFLD.^[16]^ However, the evidence of this study is of low quality. In recent years, a large number of RCTs have been added to treat NAFLD, but there is no relevant systematic review / meta-analysis.

Therefore, this study designed a protocol for systematic and meta-analysis to comprehensively evaluate the efficacy and safety of TCM in the treatment of NAFLD.

## 2 Methods

This protocol for systematic review and meta-analysis was conducted according to the PRISMA-P guidelines.^[17]^

### 2.1 Criteria for inclusion

These standards are pre-designated according to the PICOS standard, which involves patients or populations, interventions, comparisons, results, and study design.

### 2.2 Types of participants

We will include patients with NAFLD irrespective of gender, race, age, and setting. We excluded patients with any signs of mental illness or organic disease.

### 2.3 Types of interventions

It will include trials using herbs alone or in combination with traditional therapies. Chinese herbal medicine includes a single Chinese herbal medicine, a prescription consisting of several Chinese herbal medicines and herbal products extracted from natural herbs. The administration method, administration form, administration dose, and administration time are not limited.

### 2.4 Types of comparator(s)/control

The control group, accepted with sham TCM, placebo control or other active therapies, will be included. Active therapies include drugs such as antibiotics, analgesics, and corticosteroids.

### 2.5 Types of outcome indicators

#### 2.5.1 Primary outcomes

1. Aspartate aminotransferase
2. Alanine aminotransferase

#### 2.5.2 Secondary outcomes

We also considered the following outcome measures: disappearance of radiological steatosis, NAFLD fibrosis score, the Fibrosis-4 test, the BARD index, the AST-to-platelet ratio, the FibroMeter, and the FibroTest.

### 2.6 Types of studies

RCT will be included. Multiple arms trials met the above criteria will be included. For crossover trials, data will be extracted from the first period only, to avoid potential carryover effects. Another study design will be excluded.

### 2.7 Search methods for identification of studies

#### 2.7.1 Electronic searches

We will search the following databases from the inception dates to July 1, 2020: PubMed, Embase, the Cochrane Library, China National Knowledge Infrastructure, WanFang, China Science and Technology Journal Database, and China Biomedical Literature databases. The searching strategy of PubMed is presented in Table 1.

**Table 1:**
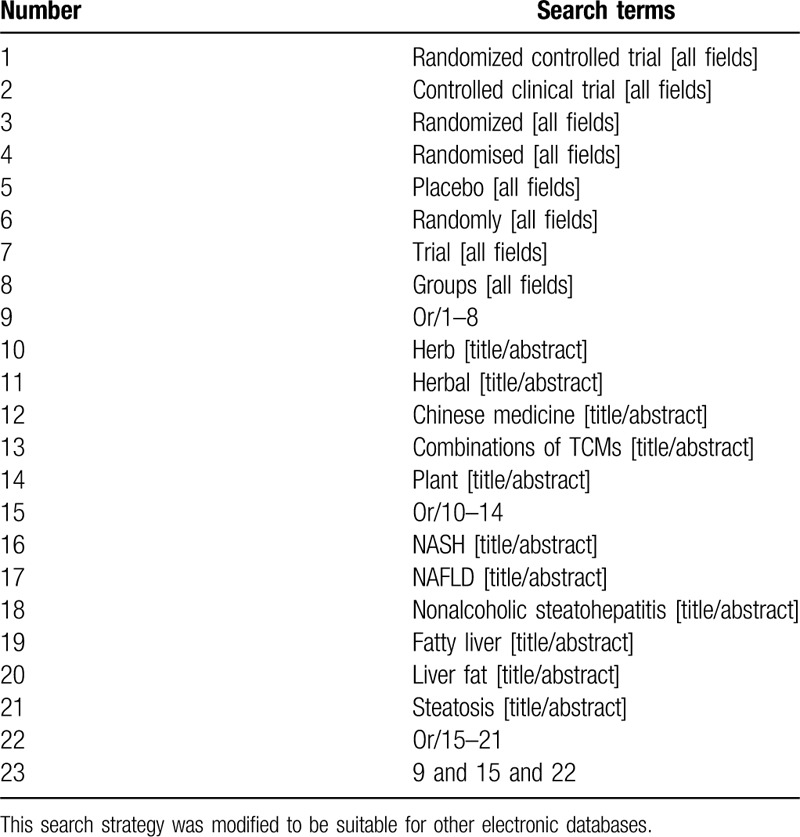
Search strategy used in PubMed database.

#### 2.7.2 Searching other resources

We will search the reference lists of the included articles and related-systematic review or meta-analysis to ensure a comprehensive search.

### 2.8 Data collection and analysis

#### 2.8.1 Selection of studies

Endnote software (X9 version) will be used to manage the research of electronic retrieval and data obtained from other sources. First, we will get rid of repeated display of author, title, and abstraction (same content in different languages or different publication formats, or 2 articles writing the same experiment from different aspects), title and abstract will be independently screened by 2 reviewers for potential The dissatisfied selection criteria for qualified research and excluded research. If reviewers cannot identify the research based on the title and abstract, they will screen the full text. When the reviewers have inconsistent opinions, they will be resolved through discussion. If no agreement is reached, the third reviewer will be consulted. The process and results of studies selection will be presented in a flow chart with Supplemental Digital Content (Appendix 1).

#### 2.8.2 Data extraction and management

Before data extraction, we will use Excel 2016 to confirm a standard data extraction form, which includes the following information: basic information (including publication, first author, publication source, etc), characteristics of the experiment (in design research, many organizations and Participants, randomized methods, blinding, analytical methods, research goals, etc), participants (age, gender, race, country, diagnosis, duration, etc), intervention and control (method intervention, Intervention, treatment, frequency of treatment, duration of 1 session, name and type of control, additional treatment, etc), outcome measurement (primary and secondary results, evaluation time, follow-up time, etc), outcome (mean, SD, intervention Later observed events, total sample size, etc). After the extraction, 2 reviewers cross-check the results. If there are differences, they will be resolved through the discussion of all reviewers. The third examiner will check the data entered to ensure data consistency and correct data entry errors.

#### 2.8.3 Assessment of risk of bias in included studies

The 2 reviewers will use the Cochrane Collaboration tool to evaluate the quality of the included trials. Six aspects will be evaluated (randomly generated serial numbers, allocation concealment, blinding of participants and personnel, blinding of results evaluation, incomplete result data, selective reports, and other deviations as needed).^[18]^ The trial will be rated for every aspect of high, low risk, or ambiguous bias. Trials rated high risk of bias in 1 or more areas will be rated high risk, while trials rated low risk of bias in all aspects will be rated low risk. If there is an unclear risk of bias in all major areas, the trial will be rated as an unclear risk. The scoring results will be checked repeatedly and the differences will be resolved through the discussion of all reviewers.

#### 2.8.4 Measures of treatment effect

All data will be synthesized using RevMan5.2 or STATA software. The 95% confidence interval of the risk ratio / odds ratio will give the results of the dichotomous data analysis, while the continuous results will use the 95% confidence interval of the mean difference / standardized mean difference Investigate.

#### 2.8.5 Dealing with missing data

We will contact the author to obtain the original data. If the author cannot be contacted or the missing data is lost, this study will be excluded and the remaining studies will be synthesized.

#### 2.8.6 Assessment of heterogeneity

We will use the Chi-squared to assess the statistical heterogeneity, if the *P* value less than .10 will be considered significant, according to the Cochrane Handbook.^[19]^ Moreover, the I^2^ value using RevMan V.5.2 will be used to quantify the impact of the statistical heterogeneity on the meta-analysis. A rough guide to the interpretation of I^2^ is as follows: 0% to 40%: might not be important; 30% to 60%: may represent moderate heterogeneity; 50% to 90%: may represent substantial heterogeneity; 75% to 100%: considerable heterogeneity.^[19]^ Besides, the importance of the observed value of I^2^ depends on 2 aspects as follows:

(1) strength of evidence for heterogeneity;
(2) magnitude and direction of effects (e.g., *P*-value from the Chi-squared test, or a confidence interval for I^2^).^[19]^

#### 2.8.7 Data synthesis

Before integrating the data, we will unify the unit of each result of different experiments according to the international unit system. Then import clinical data into RevMan software (V.5.2) for data synthesis. When I^2^ <75% comes from the heterogeneity test, the data will be synthesized and analyzed. When the heterogeneity test shows slight or no statistical heterogeneity in these trials (I^2^ value is not less than 40%), we will use a fixed-effects model for the combined data. When significant heterogeneity is detected (I^2^ 40%, <75%), a random effects model will be used for data synthesis. If there is considerable heterogeneity in the trial, no meta-analysis will be performed. In this case, we will try to determine the source of heterogeneity from both clinical and methodological aspects and will provide a qualitative summary. When more than 10 trials are included, a funnel chart will be generated to observe the report deviation.

#### 2.8.8 Subgroup analysis and meta-regression

If enough trials are included, we will use STATA software to explore the following possible sources of heterogeneity by performing subgroup analysis or meta-regression on changes in trial participant characteristics, TCM treatment, sample size, methodology, missing data, etc.

#### 2.8.9 Sensitivity analysis

Sensitivity analysis will be used to check the stability of major decisions made during the review process. Several decision nodes will be considered in the system review process, such as small sample size, lack of method, and lack of data. The results of the sensitivity analysis will be presented in the form of a summary table. As the sensitivity analysis results show, the risk of bias in the review process will be discussed.

#### 2.8.10 Evidence quality evaluation

We will use the Grading of Recommendations Assessment, Development and Evaluation system (GRADE) system to assess the quality of evidence for each outcome.^[20]^ According to the GRADE rating standards, the evidence quality will be rated with “high”, “moderate”, “low” or “very low”. The evaluation of evidence quality is mainly based on the following 5 aspect: the risk of bias of included studies, inconsistency of different research, indirectness of evidence, imprecision of results, publication bias of RCTs, large effect of sample, dose response of TCM, and all plausible confounding.^[20,21]^ The results of GRADE system evaluate will be summarized with a table to presented in the final report.^[21]^

#### 2.8.11 Ethics and dissemination

This review does not require ethical approval, because we do not endanger personal privacy or damage their rights to the data. The results of a review provide systematic perspectives and evidence for TCM for NAFLD, and will also provide inspiration for clinical practice and further research. The establishment of this study may be published in peer-reviewed journals or distributed in relevant meetings.

## Author contributions

Manman Qin and Fuqiang Yuan conceived the review protocol and drafted the manuscript. Jiankun Ren and Zhenhai Chi revised the study design and manuscript. All authors have read and approved the publication of the protocol.

**Writing – original draft:** Manman Qin and Fuqiang Yuan.

**Writing – review and editing:** Jiankun Ren and Zhenhai Chi.

## Supplementary Material

Supplemental Digital Content
